# A Western-Fed Diet Increases Plasma HDL and LDL-Cholesterol Levels in ApoD–/– Mice

**DOI:** 10.1371/journal.pone.0115744

**Published:** 2014-12-30

**Authors:** Kamilah Ali, Ehab M. Abo-Ali, M. D. Kabir, Bethany Riggins, Susanna Nguy, Lisa Li, Ujala Srivastava, Su Mya Mya Thinn

**Affiliations:** 1 The City College of New York, Biology Department, New York, New York, United States of America; 2 Graduate Center at CUNY, New York, New York, United States of America; University of Amsterdam Academic Medical Center, Netherlands

## Abstract

**Objective:**

Plasma apolipoprotein (apo)D, a ubiquitously expressed protein that binds small hydrophobic ligands, is found mainly on HDL particles. According to studies of human genetics and lipid disorders, plasma apoD levels positively correlate with HDL-cholesterol and apoAI levels. Thus, we tested the hypothesis that apoD was a regulator of HDL metabolism.

**Methods & Results:**

We compared the plasma lipid and lipoprotein profiles of wild-type (WT) C57BL/6 mice with apoD−/− mice on a C57BL/6 background after receiving a high fat-high cholesterol diet for 12 weeks. ApoD−/− mice had higher HDL-cholesterol levels (61±13-apoD−/− vs. 52±10-WT-males; 37±11-apoD−/− vs. 22±2 WT-female) than WT mice with sex-specific changes in total plasma levels of cholesterol and other lipids. Compared to WT, the HDL of apoD−/− mice showed an increase in large, lipid-rich HDL particles and according to size various quantities and sizes of LDL particles. Plasma levels of lecithin:cholesterol acyltransferase in the control and apoD−/− mice were not different, however, plasma phospholipid transfer protein activity was modestly elevated (+10%) only in male apoD−/− mice. An *in* *vivo* HDL metabolism experiment with isolated Western-fed apoD−/− HDL particles showed that female apoD−/− mice had a 36% decrease in the fractional catabolic rate of HDL cholesteryl ester. Hepatic SR-BI and LDLR protein levels were significantly decreased; accordingly, LDL-cholesterol and apoB levels were increased in female mice.

**Conclusion:**

In the context of a high fat-high cholesterol diet, apoD deficiency in female mice is associated with increases in both plasma HDL and LDL-cholesterol levels, reflecting changes in expression of SR-BI and LDL receptors, which may impact diet-induced atherosclerosis.

## Introduction

Cardiovascular disease (CVD) is the leading cause of death in Western countries. Plasma low-density lipoprotein-cholesterol (LDL-C) and high-density lipoprotein (HDL)-C levels correlate positively and negatively with CVD risk respectively [1], [2]. Although statins reduce LDL-C and with it the incidence of CVD, new therapeutic options are needed to raise plasma HDL-C in ways that are atheroprotective. CETP-inhibitors raise circulating plasma HDL-C levels, but to date none have prevented CVD [3]. Thus, the mechanisms by which HDL-C levels are increased are likely to be more relevant to atheroprotection than the actual HDL-C-raising itself [4]. The surface of HDL particles is approximately 85% protein and the HDL proteome is heterogeneous [5]. HDL-proteins occur as specific clusters in HDL subclasses, each exerting distinct biological functions–including regulation of cholesterol efflux, and anti-inflammatory, anti-oxidative, anti-thrombotic, and vasodilatory activities [6], [7]. Consequently, investigating the biological effects of HDL-associated proteins on the function of this lipoprotein is a key to understanding how HDL reduces CVD risk.

Apolipoprotein D (ApoD) is a 29-kDa glycoprotein associated mainly with plasma HDL and to a lesser extent with LDL and very low-density lipoproteins (VLDL) [8], [9]. The impact of apoD on lipid metabolism was partially clarified by recent studies revealing that apoD regulates triglyceride metabolism. Hepatic over-expression of mouse apoD reduced plasma triglyceride levels by increasing lipoprotein lipase (LPL) activity and the catabolism of triglyceride-rich particles [10]. Moreover, ApoD deficiency in mice is associated with reduced adipose tissue-LPL levels and hypertriglyceridemia [11]. Although these observations indicate that apoD regulates triglyceride metabolism, its role in the regulation of HDL, its major carrier, is unknown.

Human apoD levels are reduced in hypocholesterolemic diseases that result from deficiencies in proteins that modulate HDL-C levels, including Tangier disease and familial lecithin-cholesterol acytransferase (LCAT) deficiency. Both diseases are associated with lower apolipoproteins levels, an effect that was most profound for apoD [12]. In a study of single nucleotide polymorphisms in the human apoD gene of African Blacks, the Phe36Val SNP was associated with increased HDL_3_-C and apoAI concentrations in females [13]. Another study showed a strong positive correlation between apoD and apoAI levels, and HDL lipid content (cholesterol and phospholipids) in healthy male subjects [14]. The association between HDL and apoD may be linked to the biological activities of HDL-associated remodeling enzymes, such as LCAT, phospholipid transfer protein (PLTP), and paroxonases (PON). ApoD complexes with LCAT, and the presence of apoD in proteoliposome particles composed of either apoAI or CI stimulates the esterification activity of LCAT [15]. PLTP, which transfers phospholipids among lipoproteins and modulates HDL particle size, forms a complex composed of six apolipoproteins including apoD [16]. A human proteomics study revealed that apoD is preferentially found in the smaller, denser HDL_3_ subpopulation and is modestly positively correlated with the levels of PON1 and 3, proteins localized to small HDL_3_ [6]. The functional relationships between apoD and HDL-associated enzymes, PON and PLTP, are unknown. Collectively, however, these data suggest that apoD may regulate both HDL-C levels and its functionality, although no studies have determined the mechanism by which apoD influences plasma HDL-cholesterol. Since apoD is often up-regulated under stress conditions or disease states, mice were fed an atherogenic Western diet [8], [17], [18]. We tested the hypothesis that apoD affects circulating plasma HDL-cholesterol levels by altering the lipid and protein content of HDL and the catalytic activities of associated enzymes.

## Materials and Methods

### Mice

In-house 6–8 week old-aged matched male and female C57BL-6 and apoD−/− mice were fed a Western Diet (Harlan-TD88137) for 12 weeks. Mice had ad-libitum access to water. Animals were weighed and bled monthly after an overnight fast. At time of sacrifice, mice were fasted overnight and anesthetized with a ketamine/xylazine cocktail. Plasma and serum were collected via the retro-orbital plexus. Plasma, sera, and liver were stored at −80°C until further use. The City College of New York Institutional Animal Care and Use Committee approved all procedures.

### Fast Protein Liquid Chromatography (FPLC)-size exclusion analysis of pooled plasma samples

Pooled plasma (∼150 µl) from each genotype (n = 4–6) was injected onto Superose HR6 10/300 GL FPLC column (GE Healthcare). Lipoproteins were eluted with 24 mL of elution buffer [0.15 M NaCl, 1 mM EDTA, 0.2% w/v of sodium azide in 1X phosphate saline buffer (PBS)] and 0.5 mL fractions were collected at a flow rate of 0.5 mL/min.

### Plasma and Serum lipid analyses

Total cholesterol, free cholesterol, triglycerides, non-esterified fatty acids, and phospholipids levels were measured in plasma or sera and FPLC samples according to manufacturer instructions from Wako Chemicals or Infinity Triglycerides. The total cholesterol levels in FPLC fractions were measured with 2X Cholesterol E buffer. Plasma HDL-cholesterol measurements were determine after precipitation of apoB-containing lipoproteins. Briefly, plasma samples were mixed with equal volumes of dextran sulfate (20 g/L) and magnesium chloride (1 M) solution pH 7.0 and incubated for 10 minutes at room temperature followed by centrifugation for 30 minutes at 4°C. The supernatant was measured for cholesterol with the Cholesterol E kit (Wako).

### SDS-PAGE and Western Blotting

FPLC HDL fractions (25 µL), plasma (1 µL), or liver extract (25–50 µg) were reduced with β-mercaptoethanol/Laemmli buffer, separated on 4–20% mini Protean TGX gels (Bio-Rad), transferred to a PVDF membrane, and probed with primary antibodies for mouse apoAI (Abcam-ab20453), mouse apoD (Santa Cruz-sc34763), SR-BI (Novus Biologicals-NB400-104), LDLR (BioVision-3839-100), and β-actin (Thermo Scientific-MA-91399). Proteins were detected with ECL reagent (Promega) and the pixel intensities of bands were quantified by the Image J program (NIH). For total protein analysis, 10–20 µg of total protein was loaded on 4–20% mini TGX gel and gel stained with SimplyBlue SafeStain (Invitrogen) to detect apolipoproteins' levels in pooled FPLC fractions.

### Plasma activities of LCAT and PLTP

Plasma LCAT and PLTP activities were measured with the Roar Biomedicals Kits per vendor instructions.

### Ion mobility of plasma lipoproteins

The lipoprotein concentrations and particle size from pooled plasma (100 µL) were determined by gas-phase differential electrophoretic mobility method as previously described [19].

### Liver lipids measurement and relative mRNA expression

Livers (50 mg) from C57BL/6 and apoD−/− mice were homogenized in PBS with a bullet blender for 5 minutes at 4°C with zirconium oxide beads (ZrOB05 and ZrOB10-Next Advance). The homogenate was collected and diluted after centrifugation at 10,000 rpm and 4°C. An aliquot of homogenate was solubilized with an equal volume of 0.25% or 1% deoxycholate to measure cholesterol and triglyceride levels, respectively. Samples were incubated for 5 minutes at 37°C. Infinity Cholesterol or Triglycerides liquid stable reagents (ThermoScientific) were added to samples and glycerol & cholesterol standards for 5–15 minutes, and absorbance read at 500 nm. Hepatic lipid levels were normalized to total protein (BCA assay).

Total RNA was isolated from liver (50 mg) with Trizol Reagent (Invitrogen) after homogenization with ZrOB05 beads (Next Advance). cDNA was synthesized with 1 µg RNA using iScript cDNA Synthesis kit (Bio-Rad Laboratories). The relative mRNA expression was determined by real-time PCR using Taqman Fast Advanced Mastermix (Invitrogen) with Taqman Gene Expression Assay and calculated by the comparative C_T_ method, ΔΔC_T_. The following expression assays were used: apoAI (ApoaI- mm00437568_g1), apoE (Apoe-Mm01307193_g1), ATP-binding cassette transporter (Abca1- mm00442646_m1), LDL-receptor (Ldlr, mm00440169_m1), liver X receptor-α (Lxrα, mm00443451_m1), scavenger receptor B1 (Scarb1, mm00450234_m1), and sterol regulatory element binding transcription factor 2 (Srebf2, mm01306292_m1), and beta-2-microglobin (b2m-mm00437762_m1).

### HDL turnover studies

apoD−/− HDL was isolated from pooled mouse plasma after 12 weeks on a Western diet by sequential ultracentrifugation (density, 1.063<d<1.21). HDL was labeled with ^3^H-1,2,3-cholesteryl oleate (Perkin Elmer) as previously described [20] and 250,000 to 1 million counts per minute of ^3^H-CE-HDL was injected into the retro-orbital sinus of Western-fed mice under isofluroane anesthesia. Blood was collected at 5 minutes and 1, 5, 10, 24, and 48 hours, and liver and feces at 48 hours. Plasma decay curves were generated by subtracting the initial 5 min. injection point from each time point. The fractional catabolism was fitted to a biexponential curve using the WinSAAM modeling program, and FCR (per hour) was calculated from the normalized area under the fitted curve [20].

### Statistical analysis

Data are reported as mean ± SD and statistical significance was analyzed by a student's t-test using the GraphPad 6.0 software; *p*<0.05 was considered significant.

## Results

**Figure 1:**
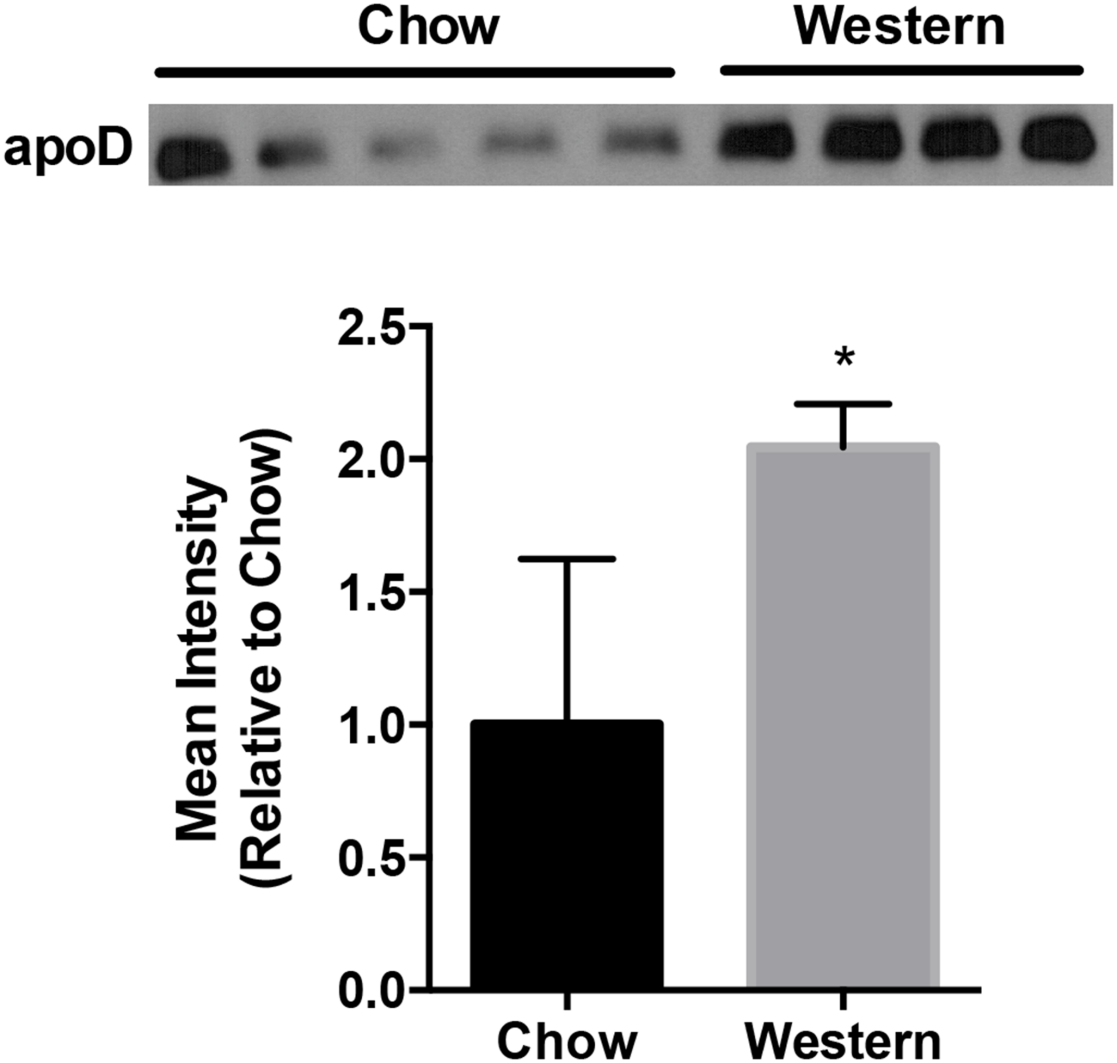
Up-regulation of plasma apoD levels in Western-fed WT mice. Western blot and semi-quantitative analyses of apoD levels in WT mice fed a 12-week chow and Western diets. Data is representative of both sexes, n = 4–5 per group, mean±SD, *p<0.05.

We investigated the role of apoD on HDL metabolism in mice on a high fat/cholesterol diet. Given that plasma expression of apoD increased 2-fold on a Western diet (WD) compared to chow diet in WT mice (Fig. 1), we analyzed plasma lipids in male and female C57BL/6 and apoD−/− mice after 12 weeks on WD diet. Following a WD, plasma HDL-C was significantly higher in both male (61±13-apoD−/− vs. 52±10-WT, p<0.001) and female apoD−/− mice (37±11-apoD−/− vs. 22±2, p<0.001) compared to WT mice (Table 1). This observation was not seen on a chow diet (data not shown). However, the commonality in HDL-cholesterol levels diverged for other lipid parameters between the sexes in apoD−/− mice (Table 1). In female apoD−/− mice, a significant increase in total cholesterol (90±10-WT vs. 140±14- apoD−/−, p<0.05) was observed due to changes in both HDL and non-HDL cholesterol levels (LDL, VLDL). Both plasma esterified cholesterol (CE) and free cholesterol (FC) were increased with the greatest change in CE (+42%). Finally, there were substantial decreases in both circulating triglyceride and NEFAs in female apoD−/− compared to control mice. In contrast, in male apoD−/− mice there were no significant differences in other plasma lipid parameters except for a decrease (0.6±0.1-apoD−/− vs. 0.7±0.1-WT, p<0.05) in non-esterified fatty acids (NEFAs). Thus, a WD dramatically impacted plasma lipids in female apoD−/− mice compared to males. In the context of these clear differences in HDL-C lipids in apoD−/− mice on an atherogenic diet, we conducted more detailed analyses of the effects of apoD deficiency on HDL content, size, and proteins.

**Table 1:** Lipid parameters in male and female WT and apoD−/− mice after 12 weeks on a Western diet.

Lipid Parameters	WT Female (n = 7–9)	apoD−/− Female (n = 7–9)	WT Male (n = 9–12)	apoD−/− Male (n = 9–12)
Total cholesterol	90±10	140±14*	174±30	178±34
HDL -C	22±2	37±11**	52±10	61±13**
Non-HDL-C	65±13	103±17**	123±35	116±28
FC	34±5	44±9*	39±6	42±7
CE	54±7	92±13****	135±28	136±30
Triglycerides	60±10	43±7**	81±15	84±9
Phospholipids	132±17	145±18	170±30	160±11
NEFA (mEq/L)	0.6±0.1	0.4±0.1*	0.7±0.1	0.6±0.1*
Body weight (g)	26±3	27±3	30±5	34±5

Non-HDL-C and cholesteryl esters (CE) are calculated values. Values represent the mean±SD, *p<0.05, **p<0.001, and ****p<0.0001. NEFA, n = 4–12. FC =  free cholesterol.

**Figure 2:**
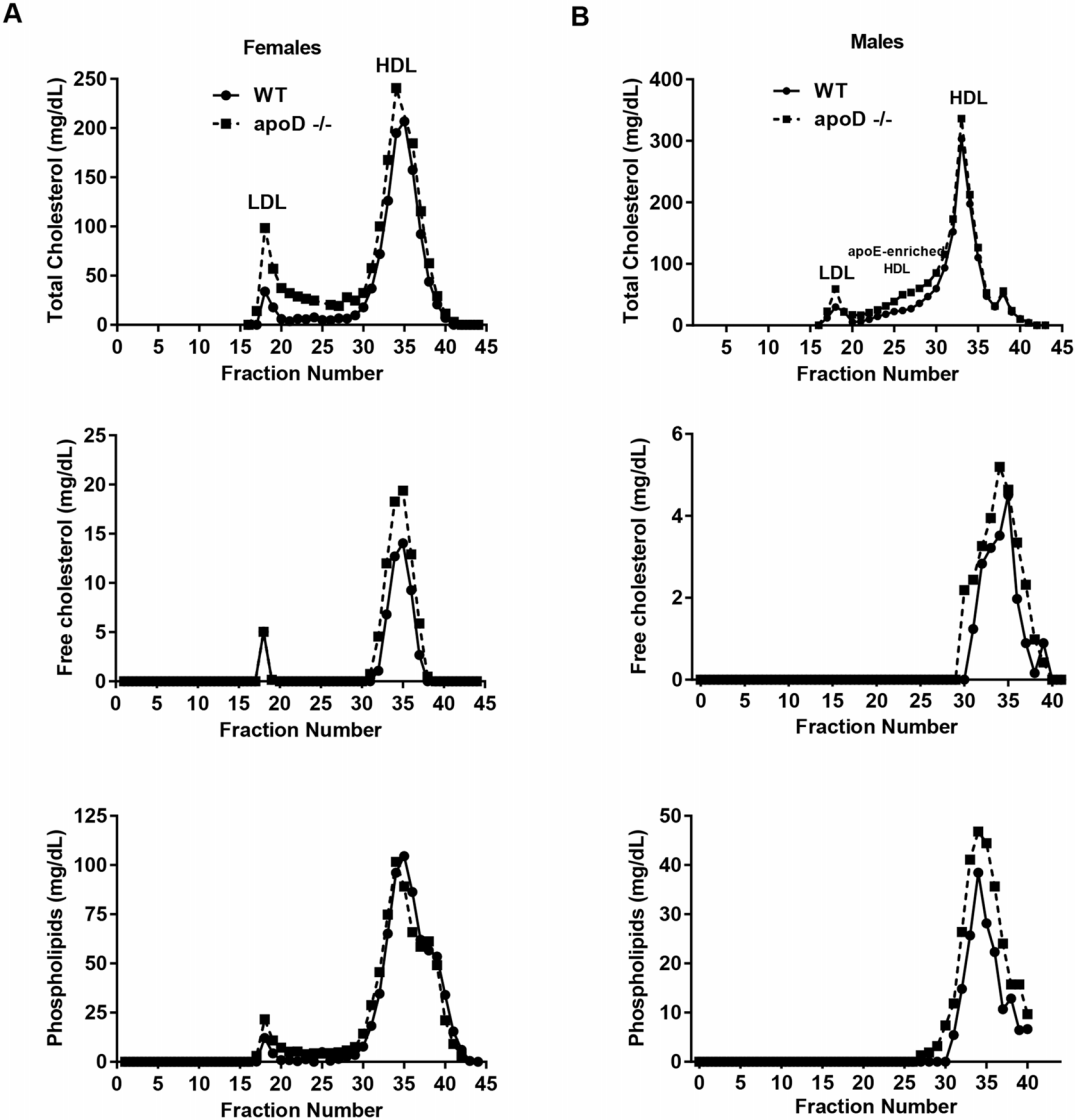
ApoE-HDL enrichment and lipids content changes in HDL particles. Size exclusion chromatography lipid profiles (total cholesterol, free cholesterol, phospholipids) of pooled plasma from A) female and B) male WT and apoD−/− mice after 12 weeks on a Western diet.

**Figure 3:**
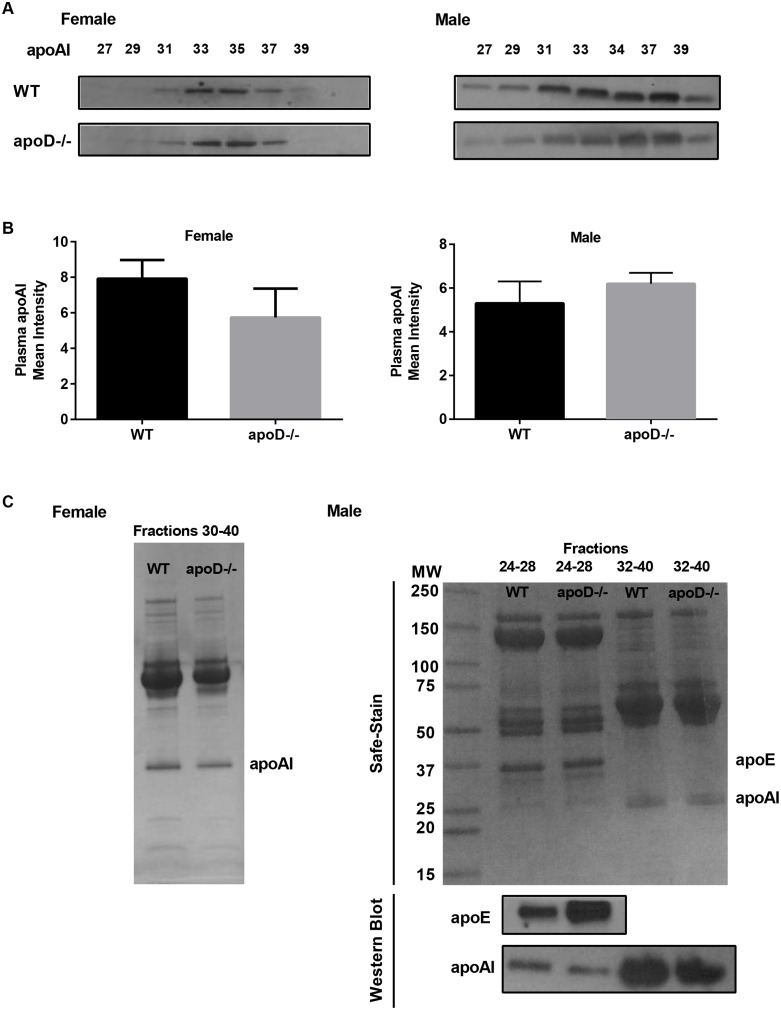
No change in plasma apoAI, but changes in apolipoproteins content in HDL-size exclusion chromatography fractions. A) Western blot analysis of apoAI levels from the SEC-HDL fractions. B) Semi-quantitative analysis of plasma apoAI levels. C) SimpleStain and Western blotting for apolipoprotein levels of pooled SEC-HDL fractions (10–20 µg).

The lipid and apolipoprotein compositions of HDL were determined in fractions isolated by size-exclusion chromatography (SEC). For female mice, apoD deficiency increased total cholesterol in both LDL and HDL fractions as well as FC in HDL particles (Fig. 2A). Semiquantitative apoAI levels from SEC-HDL fractions (31–37) were similar for both female WT and apoD−/− mice (Fig. 3A). Also, plasma apoAI levels were unchanged (Fig. 3B). The effect of apoD deficiency on lipoprotein properties in male mice was different from that of females. Size exclusion of pooled plasma, showed that compared to male WT mice, male apoD−/− mice had increased apoE-enriched HDL, which was larger as assessed by total cholesterol levels (Fig. 2B). The HDL fractions (30–40) were slightly higher in total cholesterol, free cholesterol, and phospholipids in male apoD−/− mice compared to WT (Fig. 2B). Levels of apoAI in the larger lipid-rich HDL fractions (fractions 32–40) were lower in male apoD−/− vs. WT mice (Fig. 3A). Moreover, SEC fractions 24–28 from apoD−/− mice were more apoE-rich (lane 2) but had reduced apoAI levels (lane 4) than the corresponding fractions from WT mice (lanes 1&3) (Fig. 3C). Analysis of circulating plasma levels of apoAI were not different in male apoD−/− compared to WT mice (Fig. 3B). These data suggest that apoD influences both the heterogeneity in protein and lipid content of circulating HDL-C levels with an shift towards larger, lipid rich HDL in a sex-dependent manner.

We next quantified particle sizes and lipoprotein subpopulation concentrations by ion mobility. For both sexes, there was an increase in the concentration of HDL particles of all sizes in apoD−/− versus control mice, with the majority of particles in smaller denser HDL_3/2a_. The greatest increase in concentration of HDL was, however, in larger HDL2b particles in apoD−/− mice (Table 2). The amount of the smaller particles, HDL_3_ and HDL_2a_ (76.5–105 Å), were increased by 2% in female apoD−/− mice, whereas, only a 23% increase was seen in male apoD−/− mice compared to WT mice. The larger HDL_2b_ particles (105–145 Å) were increased 28% in female mice and 69% in male apoD−/− mice. The greater increase of large HDL particles in males correlates well with the increase in apoE-HDL particles by size exclusion column analysis. Interestingly, there was also an increase in LDL particles with sex-dependent changes in the quantity and particle sizes in apoD−/− mice (Table 2). LDL particle sizes and concentrations were elevated ∼2-fold for male apoD−/− mice; however, in females there was only a 15% increase in total LDL particles, with the most profound change in very small atherogenic LDL particles (LDL4a-b) compared to WT mice. Overall, most of the circulating lipoproteins were HDL particles in apoD−/− mice, which correlate with the observed changes in circulating plasma and by size exclusion chromatography analyses.

**Table 2:** Lipoprotein particle size and concentrations (nmol/L) analyzed by IM methodology from pooled plasma of WT and apoD−/− mice fed a Western diet for 12 weeks.

Lipoprotein size fractions	Female		Male	
	WT	apoD−/−	WT	apoD−/−
**Total HDL**	11241	12488	4668	6416
Large 2b	3832	4897	1474	2493
Small 2a+b	7409	7591	3194	3923
**Total LDL**	37.21	43.32	39.68	84.12
Large 1	6.91	5.37	6.57	12.83
Medium 2a	3.12	3.56	4.25	9.57
Small 3a	6.03	5.81	6.02	14.21
Small 3b	3.05	4.04	3.89	9.00
Very small 4a	4.83	6.99	7.24	16.78
Very small 4b	8.99	13.48	11.71	21.73
**Total VLDL**	7.01	6.56	20.32	20.92
Large	0.54	0.66	7.89	6.62
Medium	2.53	2.67	7.88	8.21
Small	3.94	3.23	4.55	6.09

**Figure 4:**
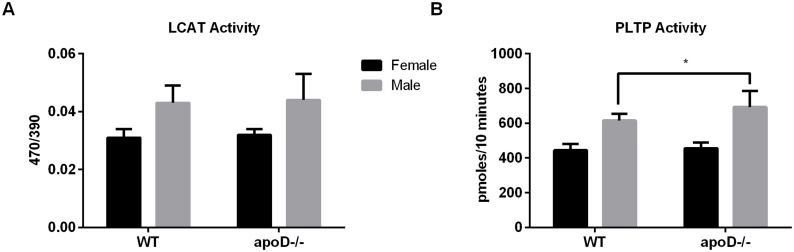
Increased PLTP activity. A) Plasma LCAT and B) PLTP activities from female and male WT and apoD−/− mice on a Western diet for 12 weeks. n = 7–14, values are mean±SD, *p<0.05.

To explain the increases in both plasma HDL-C and HDL particle size in apoD−/− mice on a Western diet, the activities of HDL-modifying proteins such as LCAT and PLTP were measured. LCAT activity was unchanged in both sexes of apoD−/− mice compared to control mice (Fig. 4A). In contrast, there was a significant but small (+10%) increase in PLTP activity in male apoD−/− mice, with no differences in females (Fig. 4B). The difference in PLTP activity may partially explain the changes in HDL particle sizes in male apoD −/− mice.

**Figure 5:**
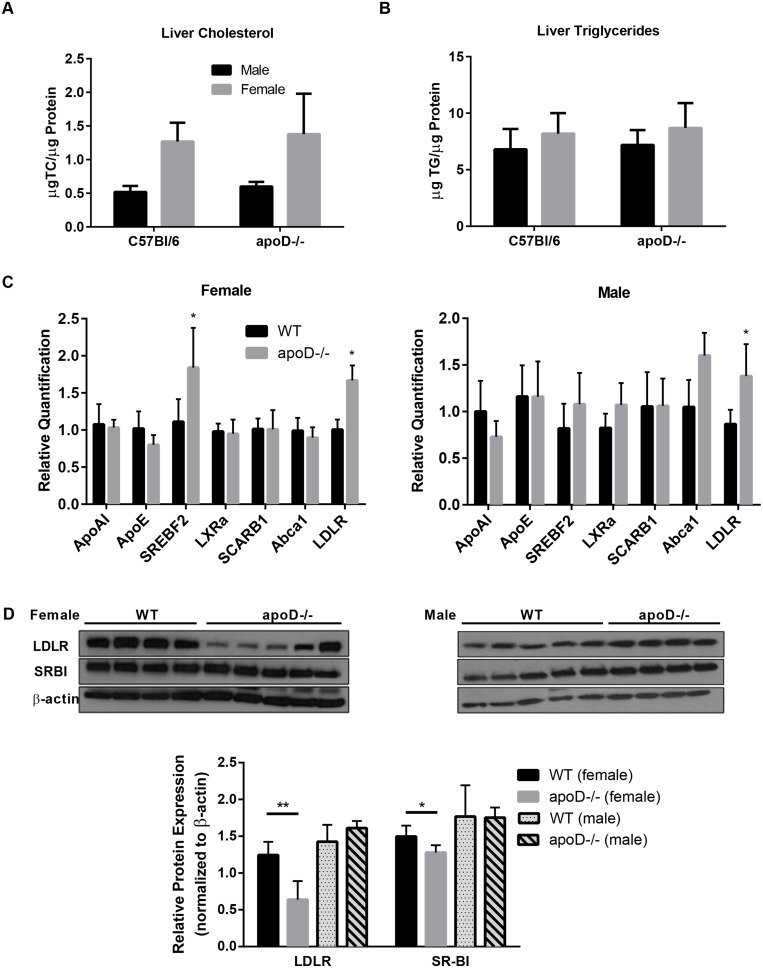
Decrease in the relative protein expression of SRBI and LDLR in female apoD−/− mice but opposite pattern for mRNA expression. Liver levels of A) Cholesterol and B) Triglyceride C) Relative expression of genes and D) Protein expression levels of LDLR and SRBI after 12 weeks on a Western diet. n = 5–7 per group, mean±SD, *p<0.05, **p<0.01.

Hepatic lipid levels and gene and protein expression were assessed to uncover a possible metabolic relationship between apoD and HDL-cholesterol levels. There were no differences found in hepatic cholesterol and triglyceride levels between control and apoD−/− mice after the 12-week atherogenic diet (Fig. 5A–B). Moreover, we considered the differential expression of genes involved in HDL biogenesis and remodeling. We found no differences in the relative expression of cholesterol transcription factor genes, sterol-regulatory element binding protein-2 (SREBF-2) and liver X receptor α (LXRα) in male mice, but in female apoD−/− mice there was a 1.5-fold increase in SREBF-2 (Fig. 5C). Furthermore, similar expression levels of apoAI and apoE were found in all mice (Fig. 5C). Finally, genes involved in hepatic HDL catabolism or synthesis, i.e., SCARB1, LDLR, and ABCA1, were analyzed; only a ∼1.5- difference in LDLR expression was observed for both sexes in apoD−/− mice (Fig. 4C). Analysis of liver protein expression of both LDLR and SR-BI receptors showed the opposite effect than the mRNA expression in female apoD−/− mice (Fig. 5D). Both plasma membrane receptors were significantly down-regulated between 15–54% (SR-BI: 1.3±0.1-apoD−/− vs. 1.5±0.2-WT, p<0.05; and LDLR: 0.6±0.3-apoD−/− vs. 1.3±0.2-WT, p<0.005) in female apoD−/− mice. There were no changes in the protein expression of both these receptors in male mice (SR-BI: 1.8±0.1-apoD−/− vs. 1.8±0.2-WT; and LDLR: 1.6±0.1-apoD−/− vs. 1.4±0.1-WT) (Fig. 5D). These findings indicate that the increase in plasma LDL-C and HDL-C in female apoD−/− mice could be attributed to a decrease in hepatic receptor protein expression, and that other HDL-associated proteins may have led to the accumulation of apoE-enriched HDL in the plasma of male apoD−/− mice.

**Figure 6:**
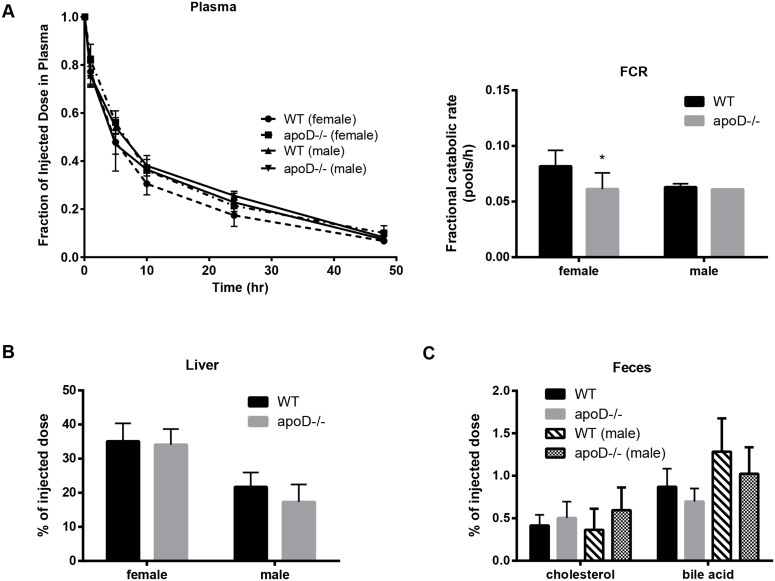
A decrease in ^3^H-CE HDL fractional catabolic rate in female apoD−/− mice only. Mice were fed a Western diet for 12 weeks and ^3^H-cholesteryl oleate-labeled apoD−/−HDL was injected. A) Changes in plasma ^3^H-CE-HDL and calculated fractional catabolic rate (FCR), B) ^3^H-cholesterol (% of injected dose) in the liver, C) ^3^H-sterol and bile acids (% of injected dose) in feces. The data represent n = 4–6 per group, mean±SD, * indicates statistical significant, p<0.05.

To determine the metabolic basis for an increase in plasma HDL levels, we investigated the catabolism of apoD−/− ^3^H-CE-HDL in mice. No substantial differences in HDL turnover or sterol tissue levels were observed in male mice (Fig. 6). In contrast, there was a 36% decrease in the HDL-CE FCR in female apoD−/− mice in comparison to WT mice (Fig. 6A, 0.082±0.014 vs. 0.061±0.014 pools/h, p<0.05). However, there were no differences observed in hepatic ^3^H-cholesterol counts or fecal extractions of ^3^H-cholesterol or bile acids in both sexes (Fig. 6B–C). Thus, down-regulation of the HDL receptor or the protein content of apoD−/−HDL particles may explain the differences in HDL catabolism and the increase in plasma HDL-levels in female apoD−/− mice.

## Discussion

This study investigated apoD as a candidate regulator of plasma HDL-C metabolism. ApoD occurs predominantly on HDL and several studies suggest that plasma apoD levels are associated with those of HDL or apoAI levels [12], [13], [14]. This is the first study to provide evidence that apoD deficiency modulates plasma HDL-C and LDL-C levels. We observed a profound increase in circulating plasma HDL-cholesterol levels and particle size in the apoD−/− mice on an atherogenic diet. These observations were confirmed by size-exclusion chromatography, as well as lipoprotein-lipid and -protein analyses. PLTP but not LCAT activity was modestly changed by apoD deficiency in male apoD−/− mice only. Despite being <5% of HDL-protein in WT mice, apoD deficiency altered the distribution of the major HDL protein and HDL particle size, which were associated with higher circulating plasma HDL-C levels [21]. More importantly, in female apoD−/− mice there was substantial decrease in plasma ^3^H-CE-HDL metabolism, which coincided with a decrease in the expression of the hepatic HDL receptor, SR-BI. These data suggest that reduction of plasma apoD levels plays a role in plasma cholesterol homeostasis.

The reduction in SR-BI protein levels may underlie the reduced FCR for HDL cholesteryl esters and thus cause elevated HDL-C levels in female vs. male apoD−/− mice. SR-BI is the key regulator of HDL-C plasma in the liver, and it mediates the selective uptake of CE from circulating HDL, leading to a reduction in HDL particle size [22]. Hepatic SR-BI deficient or SR-BI knockout mice have significantly elevated plasma HDL-C levels while overexpression of SR-BI has the opposite effect [23]–[27]. Furthermore, the plasma HDL phenotype in female apoD−/− mice resembles that of SR-BI−/− mice with increased plasma HDL-cholesterol and particle size with no changes in plasma apoA1 levels [23]. ApoD may be directly involved in regulation of SR-BI levels as SR-BI−/−: apoE−/− double knockout mice, a mouse model of coronary heart disease (CHD), had significantly higher apoD mRNA expression levels [28]. Our data show that apoD deficiency produces modest, but significant decreases in hepatic SR-BI levels in female apoD−/− mice, which could be one mechanism for a lower HDL-CE turnover rate and higher HDL plasma levels.

ApoD deficiency also modifies plasma cholesterol homeostasis via the regulation of LDL-cholesterol. In humans, apoD occurs mainly on HDL; only 5% of apoD occurs in human LDL and VLDL particles [29]. Quantitative trait loci analysis suggested that chromosome 3, specifically the apoD locus, may influence the plasma concentration of small, dense LDL-cholesterol [30]. A possible mechanism for the observed changes in LDLR expression and plasma LDL-C levels is post-transcriptional regulation since the mRNA levels of the LDLR gene were increased, but protein expression was decreased. The LDLR is regulated on a post-translational level, particularly by proprotein convertase subtilisin/kexin type 9 (PCSK9) and inducible degrader of the low-density lipoprotein receptor (IDOL). PCSK9 affects LDLR stability and trafficking by promoting its degradation by lysosomes, whereas IDOL ubiquitinates LDLR for proteasomal degradation [31]–[33]. Additional studies are needed to investigate hepatic Srebps, PCSK9, or IDOL pathways to explain the increase in LDL-C levels.

The mechanistic regulation of plasma HDL-C in male apoD−/− mice is unclear. Male apoD−/− mice showed no difference in CE-HDL catabolism, despite an increase in PLTP activity and apoE-enriched HDL particles. The increased PLTP activity in the male mice could be involved in the appearance of a minor population of spherical apoE-enriched HDL particles. Future studies will be required to determine the mechanism for changes in HDL-C levels in male apoD−/− mice.

## Conclusions

This study demonstrated that apoD may directly or indirectly modulate the levels, size, and apolipoprotein content of HDL in a sex-dependent manner. Thus, our new findings suggest that in female mice the absence of apoD may promote a pro-atherogenic phenotype via its regulation of circulating HDL-C concentrations, reflecting SR-BI deficiency, and the increase in small-dense LDL-C levels and particles. These data may support the notion that expression of apoD is protective under different stress conditions and especially in different animal models of CHD such as ischemia/reperfusion-induced myocardial infarction or diet-induced atherosclerosis [25].

## References

[1] Kannel WB, Castelli WP, Gordon T (1979) Cholesterol in the prediction of atherosclerotic disease. New perspectives based on the Framingham study. Ann Intern Med. 90:85–91. 217290 10.7326/0003-4819-90-1-85

[2] Roger VL, Go AS, Lloyd-Jones DM, Adams RJ, Berry JD, et al (2011) Heart disease and stroke stastistics-2011 update: a report from the American Heart Association. Circulation. 123:18–209. 10.1161/CIR.0b013e3182009701 PMC4418670 21160056

[3] Duivenvoorden R, Fayad ZA (2012) Safety of CETP inhibition. Curr Opin Lipidol. 26:518–524. 10.1097/MOL.0b013e32835916b3 23010697

[4] Rader DJ, Tall AR (2012) The not-so-simple HDL story: Is it time to revise the HDL. cholesterol hypothesis? Nat Med. 18:1344–1346. 22961164 10.1038/nm.2937

[5] Havel RJ, Goldstein JL, Brown MS (1980) *Lipoproteins and Lipid Transport* In: *The Metabolic Control of Disease*, 8th Edition. Saunders Publishing, Philadelphia, 398–494.

[6] Davidson WS, Silva RA, Chantepie S, Lagor WR, Chapman MJ, et al (2009) Proteomic analysis of defined HDL subpopulations reveals particle-specific protein clusters: relevance to antioxidative function. Arterioscler Thromb Vasc Biol. 229:870–876. 10.1161/ATVBAHA.109.186031 PMC2845307 19325143

[7] Camont L, Chapman MJ, Kontush A (2011) Biological activities of HDL subpopulations and their relevance to cardiovascular disease. Trends Mol Med. 17:594–603. 21839683 10.1016/j.molmed.2011.05.013

[8] Rassart E, Bedirian A, Do Carmo S, Guinard O, Sirois J, et al (2000) Apolipoprotein D. Biochim Biophys Acta. 1482:185–198. 10.1016/s0167-4838(00)00162-x 11058760

[9] McConathy WJ, Alaupovic P (1976) Studies on the isolation and partial characterization of apolipoprotein D and lipoprotein D of human plasma. Biochemistry. 15:515–520. 56198 10.1021/bi00648a010

[10] Perdomo G, Kim DH, Zhang T, Qu S, Thomas EA, et al (2010) A role of apolipoprotein D in triglyceride metabolism. J Lipid Res. 51:1298–1311. 20124557 10.1194/jlr.M001206 PMC3035493

[11] Jiménez-Palomares M, Cózar-Castellano I, Ganfornina MD, Sánchez D, Perdomo G (2011) Genetic deficiency of apolipoprotein D in the mouse is associated with nonfasting hypertriglyceridemia and hyperinsulinemia. Metabolism. 60:1767–1774. 21632073 10.1016/j.metabol.2011.04.013

[12] Alaupovic P, Schaefer EJ, McConathy WJ, Fesmire JD, Brewer HB Jr (1981) Plasma apolipoprotein concentrations in familial apolipoprotein A-I and A-II deficiency (Tangier disease). Metabolism. 30:805–809. 6790903 10.1016/0026-0495(81)90027-5

[13] Desai PP, Bunker CH, Ukoli FA, Kamboh MI (2002) Genetic variation in the apolipoprotein D gene among African blacks and its significance in lipid metabolism. Atherosclerosis. 163:329–338. 12052480 10.1016/s0021-9150(02)00012-6

[14] James RW, Martin B, Pometta D, Grab B, Suenram A (1986) Apoprotein D in a healthy, male population and in male myocardial infarction patients and their male, first-degree relatives. Atherosclerosis. 60:49–53. 3085685 10.1016/0021-9150(86)90086-9

[15] Kostner GM, Steyrer E (1988) Activation of lecithin-cholesterol acyltransferase by. apolipoprotein D: comparison of proteoliposomes containing apolipoprotein D, A-I or C-. Biochim Biophys Acta. 958:484–491. 3124886 10.1016/0005-2760(88)90235-4

[16] Albers JJ, Vuletic S, Cheung MC (2012) Role of plasma phospholipid transfer protein in lipid and lipoprotein metabolism. Biochim Biophys Acta. 1821:345–357. 21736953 10.1016/j.bbalip.2011.06.013 PMC3192936

[17] Ganfornina MD, Do Carmo S, Lora JM, Torres-Schumann S, Vogel M, et al (2008) Apolipoprotein D is involved in the mechanisms regulating protection from oxidative stress. Aging Cell. 7:506–515. 18419796 10.1111/j.1474-9726.2008.00395.x PMC2574913

[18] Dassati S, Waldner A, Schweigreiter R (2014) Apolipoprotein D takes center stage in the stress response of the aging and degenerative brain. Neurobiol Aging 35:1632–1642. 24612673 10.1016/j.neurobiolaging.2014.01.148 PMC3988949

[19] Caulfield MP, Li S, Lee G, Blanche PJ, Salameh WA, et al (2008) Direct determination of lipoprotein particle sizes and concentrations by ion mobility analysis. Clin Chem. 54:1307–1316. 18515257 10.1373/clinchem.2007.100586

[20] Tietge UJ, Maugeais C, Cain W, Grass D, Glick JM, et al (2000) Overexpression of secretory phospholipase A(2) causes rapid catabolism and altered tissue uptake of high density lipoprotein cholesteryl ester and apolipoprotein A-I. J Biol Chem. 275:10077–10084. 10744687 10.1074/jbc.275.14.10077

[21] Albers JJ, Cheung MC, Ewens SL, Tollefson JH (1981) Characterization and immunoassay of apolipoprotein D. Atherosclerosis. 39:395–409. 10.1016/0021-9150(81)90025-3 6789837

[22] Acton S, Rigotti A, Landschulz KT, Xu S, Hobbs HH, et al (1996) Identification of scavenger receptor SR-BI as a high density lipoprotein receptor. Science. 271:518–520. 8560269 10.1126/science.271.5248.518

[23] Rigotti A, Trigatti BL, Penman M, Rayburn H, Herz J, et al (1997) A targeted mutation in the murine gene encoding the high density lipoprotein (HDL) receptor scavenger receptor class B type I reveals its key role in HDL metabolism. Proc Natl Acad Sci USA. 94:12610–12615. 9356497 10.1073/pnas.94.23.12610 PMC25055

[24] Varban ML, Rinninger F, Wang N, Fairchild-Huntress V, Dunmore JH, et al (1998) Targeted mutation reveals a central role for SR-BI in hepatic selective uptake of high density lipoprotein cholesterol. Proc Natl Acad Sci U S A. 95:4619–4624. 9539787 10.1073/pnas.95.8.4619 PMC22539

[25] Brundert M, Ewert A, Heeren J, Behrendt B, Ramakrishnan R, et al (2005) Scavenger Receptor Class B Type I Mediates the Selective Uptake of High-Density Arterioscler Thromb Vasc Biol. 25:143–14. 10.1161/01.ATV.0000149381.16166.c6 15528479

[26] Ueda Y, Gong E, Royer L, Copper PN, Francone OL, et al (2000) Relationship between expression levels and atherogenesis in scavenger receptor class B, type I transgenics. J. Biol. Chem. 275:20368–20373. 10.1074/jbc.M000730200 10751392

[27] Wang N, Arai T, Ji Y, Rinninger F, Tall AR (1998) Liver-specific overexpression of scavenger receptor BI decreases levels of very low density lipoprotein ApoB, low density lipoprotein ApoB, and high density lipoprotein in transgenic mice. J. Biol. Chem. 273:32920–32926. 10.1074/jbc.273.49.32920 9830042

[28] Tsukamoto K, Mani DR, Shi J, Zhang S, Haagensen DE, et al (2013) Identification of apolipoprotein D as a cardioprotective gene using a mouse model of lethal atherosclerotic coronary artery disease. Proc Natl Acad Sci U S A. 110:17023–17028. 24082102 10.1073/pnas.1315986110 PMC3801016

[29] Camato R, Marcel YL, Milne RW, Lussier-Cacan S, Weech PK (1989) Protein polymorphism of a human plasma apolipoprotein D antigenic epitope. J Lipid Res. 30:865–875. 2477480

[30] Rainwater DL, Almasy L, Blangero J, Cole SA, VandeBerg JL, et al (1999) A genome search identifies major quantitative trait loci on human chromosomes 3 and 4 that influence cholesterol concentrations in small LDL particles. Arterioscler Thromb Vasc Biol. 19:777–783. 10073986 10.1161/01.atv.19.3.777

[31] Kwon HJ, Lagace TA, McNutt MC, Horton JD, Deisenhofer J (2008) Molecular basis for LDL receptor recognition by PCSK9. Proc Natl Acad Sci USA. 105:1820–1825. 18250299 10.1073/pnas.0712064105 PMC2538846

[32] Zhang DW, Lagace TA, Garuti R, Zhao Z, McDonald M, et al (2007) Binding of proprotein convertase subtilisin/kexin type 9 to epidermal growth factor-like repeat A of low density lipoprotein receptor decreases receptor recycling and increases degradation. J Biol Chem. 282:18602–18612. 17452316 10.1074/jbc.M702027200

[33] Zhang L, Reue K, Fong LG, Young SG, Tontonoz P (2012) LDLR Axis -IDOL-Feedback Regulation of Cholesterol Uptake by the LXR. Arterioscler Thromb Vasc Biol. 32:2541–2546. 22936343 10.1161/ATVBAHA.112.250571 PMC4280256

